# Phospholipids and protein adaptation of *Pseudomonas* sp. to the xenoestrogen tributyltin chloride (TBT)

**DOI:** 10.1007/s11274-014-1659-3

**Published:** 2014-05-03

**Authors:** Przemysław Bernat, Paulina Siewiera, Adrian Soboń, Jerzy Długoński

**Affiliations:** Department of Industrial Microbiology and Biotechnology, Faculty of Biology and Environmental Protection, University of Lodz, Banacha 12/16, 90-237 Lodz, Poland

**Keywords:** Organotins, Tributyltin, *Pseudomonas*, Phospholipids, Proteomics

## Abstract

A tributyltin (TBT)-resistant strain of *Pseudomonas* sp. isolated from an overworked car filter was tested for its adaptation to TBT. The isolate was checked for organotin degradation ability, as well as membrane lipid and cellular protein composition in the presence of TBT. The phospholipid profiles of bacteria, grown with and without increased amounts of TBT, were characterized using liquid chromatography/electrospray ionization/mass spectrometry. The strain reacted to the biocide by changing the composition of its phospholipids. TBT induced a twofold decline in the amounts of many molecular species of phosphatidylglycerol and an increase in the levels of phosphatidic acid (by 58 %) and phosphatidylethanolamine (by 70 %). An increase in the degree of saturation of phospholipid fatty acids of TBT exposed *Pseudomonas* sp. was observed. These changes in the phospholipid composition and concentration reflect the mechanisms which support optimal lipid ordering in the presence of toxic xenobiotic. In the presence of TBT the abundances of 16 proteins, including TonB-dependent receptors, porins and peroxidases were modified, which could indicate a contribution of some enzymes to TBT resistance.

## Introduction

Tributyltin (TBT) is a xenoestrogen, which disrupts the normal function of human and animal hormonal systems by modelling their activity. Endocrine disruption and disruption of lipid homeostasis are the main toxic effects (Pagliarani et al. 2013). Among organisms attacked by TBT, bacterial cells are particularly useful to study the molecular toxicity of membrane-active compounds, offering advantages over eukaryotic cells owing to the simple membrane organization (Martins et al. 2005). TBT has a negative impact on bacterial growth, chemotaxis, respiration, and membrane physical properties in microbial strains (Martins et al. 2005). Therefore, in order to cope with this threat, organotin-resistant bacteria have developed adaptive responses to the toxicity of TBT. Though many authors have reviewed the proposed hypothesis, little is known about the mechanism of resistance of microorganisms to organotins (Dubey and Roy 2003; Fukushima et al. 2012). Among TBT-tolerant microorganisms, strains from the genus *Pseudomonas* are often mentioned (Roy and Nair 2007). It is also suggested that gene PA0320 in *Pseudomonas aeruginosa* 25 W as well as other genes such as a transglycosylase homologue in *Alteromonas* sp. and a multidrug efflux transporter in *Pseudomonas stutzeri* could be involved in stress tolerance against TBT (Fukushima et al. 2012). However, studies on the adaptive stress responses of TBT-resistant bacteria including changes in membrane lipid and protein composition are still missing.

In this study, a systematic analysis of growth rates, degradation efficiency, regulation of cellular protein and membrane lipid composition of *Pseudomonas* cultivated with TBT was carried out in order to elucidate cell adaptation to the organotin substrate. This knowledge will give some insights into the understanding of the parameters involved in adaptive resistance acquisition by *Pseudomonas* species.

## Materials and methods

### Chemicals

Organotins were purchased from Sigma–Aldrich. 1,2-Dimyristoyl-*sn*-glycero-3-phospho-*rac*-(1-glycerol) (sodium salt) (14:0/14:0 PG), 1,2-dilauroyl-*sn*-glycero-3-phosphoethanolamine (12:0/12:0 PE) and 1,2-dimyristoyl-*sn*-glycero-3-phosphate (sodium salt) (14:0/14:0 PA) were purchased from Avanti Polar Lipids. All of these compounds were added to a solution of mixed internal standards at the concentration of 0.1 mg ml^−1^ (IS solution). The other chemicals were from J. T. Baker, Fluka and POCh (Gliwice, Poland). All the chemicals were high purity grade reagents. Stock solutions of TBT were prepared at 5 mg ml^−1^ ethanol.

### Microorganisms and growth conditions

For this study, *Pseudomonas* strains isolated from polluted environments were used. Based on the results of research concerning *Pseudomonas* tolerance to TBT (Fukushima et al. 2012), it was expected that our study would allow us to find a microorganism with high organotin-resistance.

Five-day-old bacterial cultures on malt extract agar slants were used to inoculate 20 ml universal medium (per liter: 1.3 g yeast extract, 15 g peptone, 5 g glucose) (in 100-ml Erlenmeyer flasks). The cultivation was carried out on a rotary shaker (140 rev min^−1^) for 48 h at 28 °C. Two ml of the homogenous preculture was introduced into 18 ml of medium with TBT (at the concentrations of 10, 20, 30 and 40 mg l^−1^) or without the organotin in the control cultures (in 100 ml flasks). The cultures were incubated for 7 days at 28 °C on a rotary shaker (140 rev min^−1^). Although *Pseudomonas* sp. B-219 is a psychrophilic strain, the temperature of 28 °C was recommended by DSMZ-German Collection of Microorganisms for incubation of *P. proteolytica* (http://www.dsmz.de). Moreover, the growth inhibition was not observed at 28 °C. Bacterial biomass was separated from culture media by filtering through a Sartorius filter (0.22 µm) and then dried at 105 °C to reach a constant weight. The specific growth rate (µ) was calculated by the least-squares fitting to the linear part of the semilogarithmic plot of bacterial biomass versus time.

### Phospholipid extraction procedure

Bacterial biomass from the stationary phase of growth (the samples were withdrawn from 120-h-old cultures) was separated from culture media by centrifugation at 4,000×*g*. It was homogenized (Misonix Sonicator) with 10 ml of a CHCl_3_–MeOH mixture (2:1, v/v). A total of 30 µl of IS solution was poured into each sample before extraction. To the crushed cells 2 ml of 0.8 % NaCl was added, the vials were vortexed for 1 min and centrifuged. The lower organic phase was collected, treated with anhydrous sodium sulphate, and evaporated under reduced pressure. The residue was then re-dissolved in 2 ml of methanol/CHCl_3_ (4:1, v/v) and stored at −20 °C pending analysis.

### Determination of PL molecular species by LC–MS/MS

Measurement of phospholipids was carried out according to our previous procedure (Bernat et al. 2013).

Identification as well as quantification of lipid molecular species were performed using precursor ion scanning (PIs) and multiple reaction monitoring (MRM), respectively. An information-dependent acquisition (IDA) method, PIs → EPI, was used. The spectra were obtained over a range from m/z 100 to 900. A scan of the precursor for m/z 153 was used to detect the subspecies of PLs. Then, a comprehensive list of MRM transitions was generated to follow the fatty acyl compositions of the studied lipids.

### Organotin analysis

The organotin content in bacterial cultures were determined according to the procedure of Bernat et al. (2009).

### Protein profile

Bacterial biomass from the stationary phase of growth was separated from culture media by centrifugation at 7,000×*g*, washed with a lysis buffer and centrifuged. Then, the biomass was homogenized using a Fast-Prep 24 instrument (MP Biomedicals). The samples were centrifuged (10,000×*g*) and the cell supernatant was separated from the cell pellet. The proteins in the supernatant were precipitated with 20 % TCA (20 % w/v) at 4 °C for 45 min and then centrifuged (10,000×*g*) for 20 min. Protein extracts were washed twice with cold acetone and centrifuged. Next, protein precipitates were transferred to Eppendorf vials (1.5 ml) and the rest of acetone was evaporated at 40 °C for 2 min. The protein residue was suspended in 30 μl of 0.2 M NaOH for 2 min and made up to the volume of 500 µl with the SSSB buffer.

### One-dimensional electrophoresis

SDS-PAGE mini gels (12.5 %) were prepared according to the procedure described in the instruction supplied together with the set for electrophoresis (Mini-PROTEAN Tetra Cell (Bio-Rad)). The protein content of each sample was diluted to the same amount of protein using the SSSB buffer. Next, the samples were mixed with Laemmli Sample Buffer at the ratio 1:1 (v/v) and incubated for 5 min at 95 °C (without a mass marker) in an Eppendorf thermoblock (Eppendorf). Then, 20 µl of each sample was applied in the gel. Electrophoresis was conducted at 60 V for the stacking gel and 90 V for the running gel. The gels were calibrated with the molecular mass marker 6,500–200,000 Da (Sigma–Aldrich) and stained with Coomassie blue. The result was documented in a gel documentation system.

### In-gel trypsin digestion

Gel bands containing protein from areas of interest were excised and placed into 1.5 ml protein LoBind tubes (Eppendorf). Gel pieces were washed with distilled water, completely destained using 50 mM NH_4_HCO_3_:acetonitrile (ACN) (50:50 v/v) and dehydrated with 100 µl of ACN. Reduction and alkylation of gel pieces were carried out by the addition of 50 µl/band of 10 mM dithiothreitol (DTT) in 100 mM NH_4_HCO_3_ (incubated at 56 °C for 30 min) and the addition of 50 µl/band of 50 mM iodoacetamide in 100 mM NH_4_HCO_3_ (30 min in dark). Gel bands were washed with 100 µl of 100 mM NH_4_HCO_3_ and then with 100 µl of ACN, (vortexed for 15 min). The band pieces were then dried. Digestion was carried out using sequencing-grade modified trypsin (20 ng/µl in 25 mM NH_4_HCO_3_, Promega). Sufficient amount of trypsin solution was added to swell the gel pieces and incubated at 37 °C, overnight. Peptides were extracted from the gel pieces by washing twice with 50 µl of 0.1 % trifluoroacetic acid (TFA) solution in 2 % ACN and vortexed for 30 min. The samples were stored at 4 °C, or the extracts were completely dried and stored at −80 °C (reconstituted in 0.1 % TFA before analysis) for a short time, and for a long time, respectively.

### LC–MS/MS

Sequencing of peptides was performed using an Eksigent ekspert™ microLC 200 system (Eksigent). The extracts (15 μl injection, loop volume 10 µl) were separated on a reversed-phase Eksigent 3C8-CL-120 column (100 × 0.5 mm, 3 μm) at 40 °C. Gradient flow rates were as follows: 28.5 µl/min A (1.5 µl/min B), 1 min 9.5 µl/min A (0.5 µl/min B), 40 min 6 µl/min A (4 µl/min B), 45 min 0.5 µl/min A (9.5 µl/min B), 50 min 0 µl/min A (20 µl/min B), 50.1 min 19 µl/min A (1 µl/min B), 53 min 9.5 µl/min A (0.5 µl/min B). Both solvents (A- H_2_O, B-ACN) were supplemented with 0.1 % of formic acid. Analyses were performed on an QTRAP 4500 LC–MS/MS system (AB Sciex) using electrospray ionization (ESI). MS/MS spectra were acquired in the data-dependent mode. Doubly, triply or fourthly charged peptide ions were identified from a survey scan (m/z 500–1,500), following which individual precursor ions were automatically selected for fragmentation.

### MALDI–TOF/TOF–MS

Tryptic digests of the protein were analyzed using an 5800 MALDI-time of flight (TOF/TOF) mass spectrometer (AB Sciex). A two-layer method was applied, in which the first layer was formed by applying 0.5 μL α-cyano-4-hydroxycinnamic acid (10 mg ml^−1^ in 50 % solution of water/ACN) (Protea), 0.5 μl of sample to the MALDI target plate and dried very quickly in air. For the second layer the procedure was repeated. The 25 most intense peaks, were automatically chosen and used for MS/MS (exclusion list containing common contaminants was also applied) The TOF MS analysis was done in the mass range 800–4,000 Da, 2,600 V/1,000 Hz laser relative energy with 2,000 shots per sample. The precursor selection order in this mode was set from the strongest to the weakest. The instrument in the TOF MS mode was externally calibrated. The TOF/TOF MS/MS analysis was conducted in the mass range 10–4,000 Da, 3,400 V/1,000 Hz laser relative power, CID gas (air) switched on at a pressure of ca 7 × 10^−7^ and up to 4,000 shots per precursor with dynamic exit. The precursor selection was set from the weakest to the strongest in this mode. The external calibration of the MS/MS mode with the fragments of Glu-fibrinopeptide (1,570.677 m/z) was applied.

### Data analysis

The most intense peptides were selected for tandem mass spectrometry (MS/MS) analysis, and the combined MS and MS/MS data were analyzed using ProteinPilot 4.5 (AB Sciex) interfaced with the Mascot search engine (Matrix Science). The data were searched against NCBInr (version 12.2013), restricted to NCBI *Pseudomonas* species database (3,730,891 proteins). MS/MS ion searches were performed with the following settings: trypsin was chosen as protein-digesting enzyme, up to two missed cleavages were tolerated, the following variable modifications were applied: Acetyl (N-term), Carbamidomethyl (C), Deamidated (NQ), Gln → pyro-Glu (N-term Q), Glu → pyro-Glu (N-term E), Oxidation (M), Phospho (ST) and Phospho (Y). Searches were done with a peptide mass tolerance of 50 ppm and a fragment ion mass tolerance of 0.3 or 0.7 and 0.3 Da for MALDI-TOF/TOF and LC–MS/MS analysis, respectively. Based on the results obtained, the peptides with the high score and percentage of coverage were taken into consideration.

The experimental data were the means of at least 3 independent experiments. The significance of differences between the control and the treatment mean values was determined by Mann–Whitney U-test.

All phospholipid peaks were integrated using the Analyst (version 1.52) software from AB Sciex. The lipids in each class were quantified against the internal standard of that class.

The Double-bond index (DBI), which indicates the unsaturation level of lipids, was calculated by the equation: DBI = [sum of (N × % lipid molecular species)]/100, where N is a number of double bonds in each lipid molecular species and % refers to % of a complex lipid class (Su et al. 2009).

## Results

### Screening of *Pseudomonas* strains for the ability to eliminate TBT (10 mg l^−1^)

**Fig. 1:**
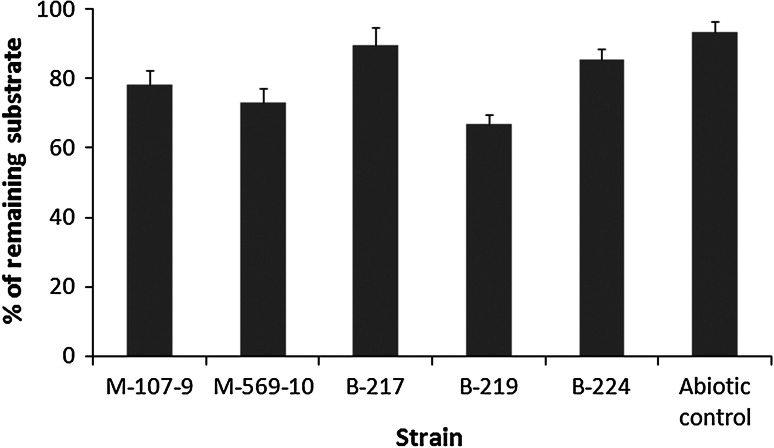
TBT removal by isolated strains in universal medium after 5 days of culturing (TBT added at 10 mg l^−1^)

TBT at the initial concentration of 10 mg l^−1^ was utilized in universal medium. The results obtained for the organotin elimination and expressed as a percentage of depletion after 5 days of incubation are presented in Fig. 1. Among the examined microorganisms, strain B-219 showed the best degradation efficiency and was selected for further study.

The 16S rRNA analysis and a comparison of the obtained sequences using the BlastN algorithm in GenBank revealed that strain B-219 is *Pseudomonas* sp. with 100 % of probability to *Pseudomonas* sp. Pi 3-8, which is closely (99.6 %, E value = 0) related to *Pseudomonas proteolytica* (Romanenko et al. 2008).

### Effect of the initial TBT concentration on *Pseudomonas* sp. B-219 growth and butyltin degradation

**Fig. 2:**
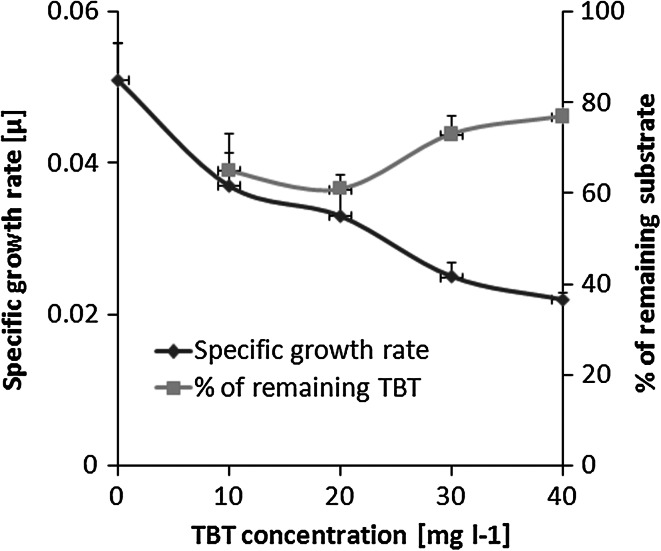
Effects of growing TBT concentrations on the specific growth rate and the organotin elimination by *Pseudomonas* sp. B-219 in universal medium after 5 days of culturing

**Fig. 3:**
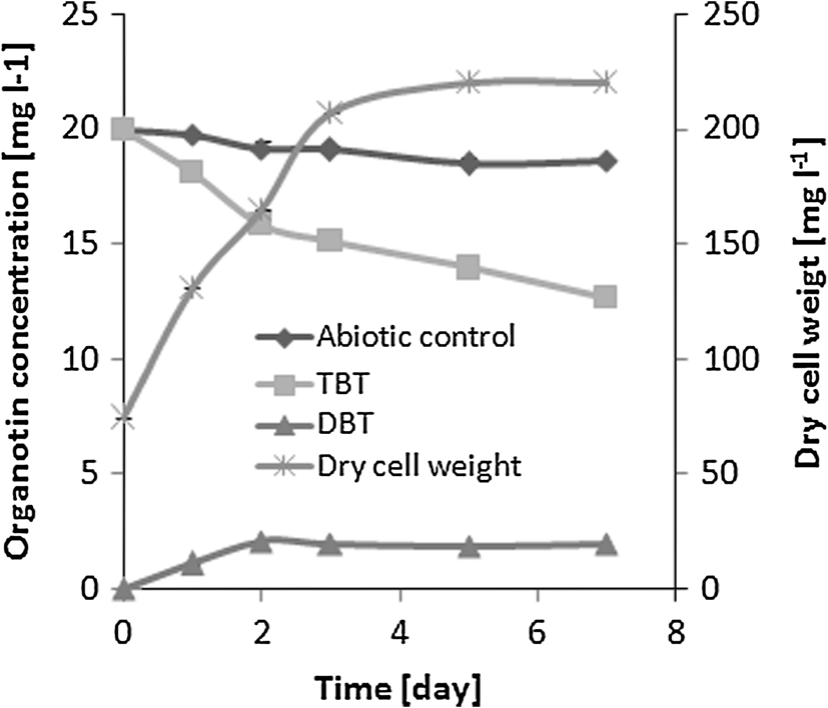
TBT degradation by *Pseudomonas* sp. B-219 and biomass formation during 7 days of incubation in the universal medium (TBT added at 20 mg l^−1^)

TBT was utilized by *Pseudomonas* sp. B-219 in universal medium with the xenoestrogen content of up to 40 mg l^−1^ (Fig. 2). However, at TBT concentrations of 30 and 40 mg l^−1^ a strong decrease in the bacterial growth rate as well as the organotin elimination efficiency was noticed at a higher TBT level. Taking into account the results obtained, in the next step, the time course of TBT (20 mg l^−1^) degradation during 7 days of incubation was examined (Fig. 3). The intensity of TBT transformation to DBT was correlated with bacterial growth.

### Phospholipid profile of *Pseudomonas* sp. B-219 in the presence of TBT

**Fig. 4:**
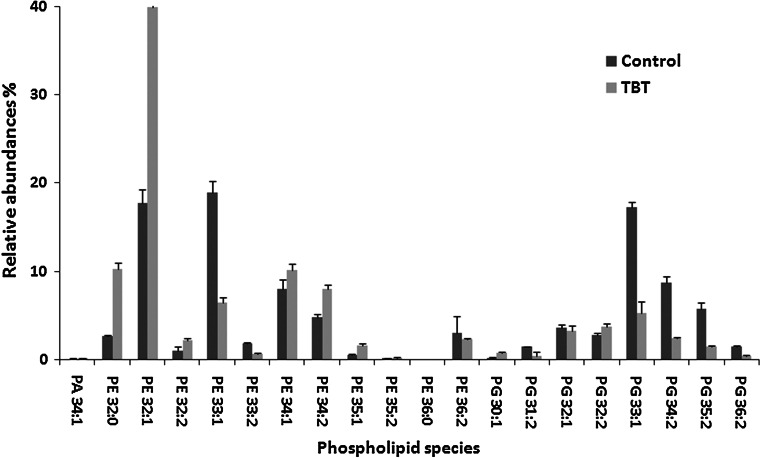
Comparison of phospholipid composition of *Pseudomonas* sp. exposed to TBT after 5 days of incubation. *PA* phosphatidic acid, *PE* phosphatidylethanolamine, *PG* phosphatidylglycerol

Phospholipids were identified based on their mass spectra in a negative ionization mode. Positions of fatty acids on the *sn* positions were not determined. Distinct species of PE, PG and PA were characterized referring to the number of carbon atoms and unsaturated carbon bonds (Fig. 4). Fatty acids in phospholipid positions *sn*-*1* and *sn*-*2* were either saturated or monounsaturated with the chain length from 14 to 19. This was in accordance with the studies of *Pseudomonas* *aerugienosa* phospholipids using the GC/MS technique (Tashiro et al. 2011).

The phospholipid composition of *Pseudomonas* sp. B-219 grown in the medium without TBT was relatively simple, and exhibited 6 major phospholipids (Fig. 4). The dominant phospholipids were PE 32:1, PE 33:1, PE 34:1, PE 34:2, PG 33:1 and PG 34:2. The PE class constituted 59 % of the total phospholipids, followed by PG and PA (41 and 0.1, respectively) (Table 1). This fraction was also the most abundant phospholipid in *Pseudomonas* species described by others (Fang et al. 2000; Tashiro et al. 2011).

**Table 1:** Phospholipid composition and double bond index determined in *Pseudomonas* sp. B-219 cells exposed to TBT (20 mg l^−1^)

	Control	TBT
*PA*		
Amount (µg/mg d.w.)	0.01 ± 0.005	0.03 ± 0.01
Relative abundance (%)	0.07 ± 0.02	0.19 ± 0.03
*PE*		
Amount (µg/mg d.w.)	10.78 ± 1.03	14.32 ± 0.8
Relative abundance (%)	58.6 ± 2.1	81.90 ± 1.1
*PG*		
Amount (µg/mg d.w.)	7.6 ± 0.55	3.13 ± 0.43
Relative abundance (%)	41.36 ± 1.19	17.91 ± 0.92
DBI	1.28 ± 0.02	1.12 ± 0.06

*PA* phosphatidic acid, *PE* phosphatidylethanolamine, *PG* phosphatidylglycerol, *DBI* double bond index

Changes in PLs resulting from the TBT presence were observed. Comparison of samples from the TBT supplemented medium and the control medium showed that the strain exposed to TBT had increased levels of PE, and PA (Table 1). On the other hand, the PG fraction, which constituted about 18 %, drastically decreased.

The PA level can increase significantly in response to different biotic and abiotic stress factors (Testerink and Munnik 2005; Darwish et al. 2009). In the present study we found that the content of PA species increased in cells exposed to high contents of TBT (Fig. 4).

The total concentration of phospholipids in TBT samples decreased slightly (from 18.4 to 17.5 µq/mg d.w., Table 1).

To identify the possible roles of phospholipid fatty acids in TBT tolerance mechanisms in *Pseudomonas* sp., we used the DBI index, which indicates the level of lipid unsaturation (Table 1). In TBT-exposed cells, DBI of PLs species was lower than in control samples.

### Analysis of TBT-induced protein

**Fig. 5:**
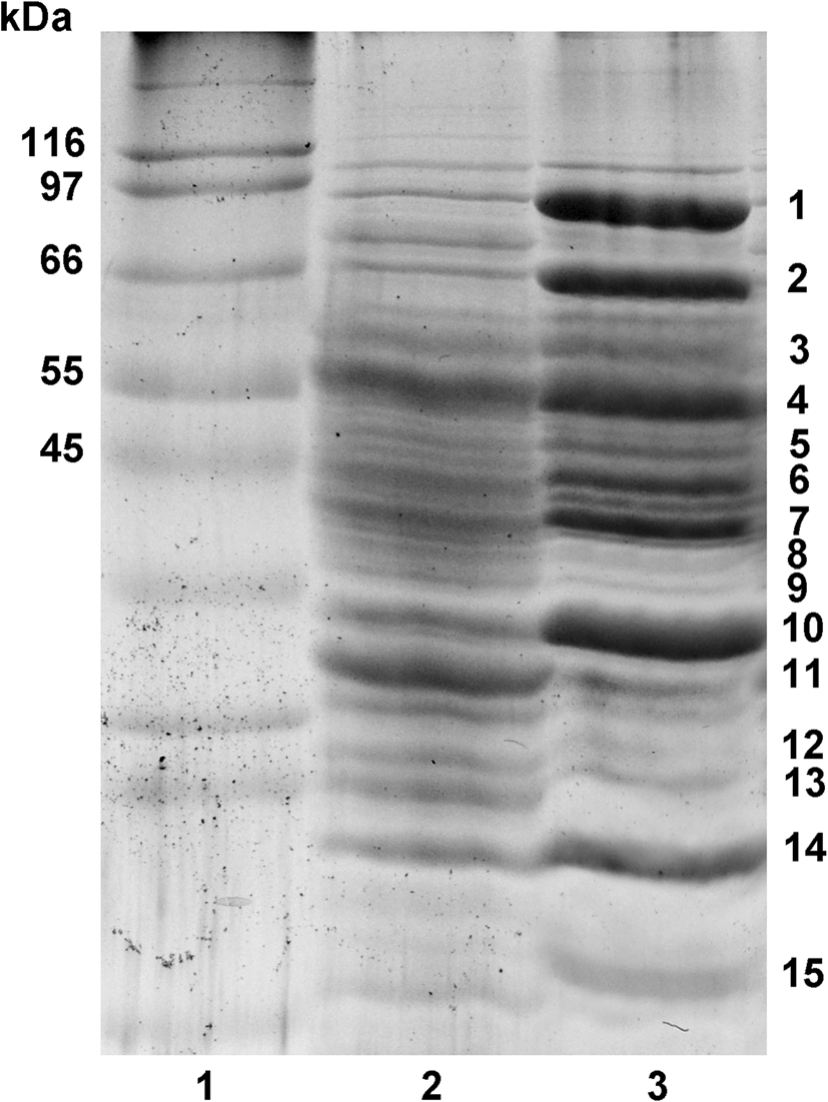
Protein profile of *Pseudomonas* sp. B-219 in the presence of TBT (20 mg l^−1^) after 5 days of culturing. 1. Protein marker, 2. protein sample of cells grown in the universal medium 3. protein sample of cells grown in universal medium + TBT. Bold point to the protein bands specific for TBT

The total protein analysis of *Pseudomonas* sp. B-219 (determined by Bradford assay) revealed that cells exposed to TBT did not inhibit the synthesis of bacterial proteins (0.145 and 0.127 mg protein/mg d.w., for control and TBT, respectively). SDS-PAGE revealed fifteen major bands, which were altered in the presence of TBT: ten of them showed increases in intensity (Fig. 5; Table 2). A total of 21 individual protein bands were excised from the gel, subjected to in-gel digestion with trypsin and the resultant peptides were analysed by MALDI–TOF–MS and LC–MS/MS (Table 2). Using Protein Pilot and Mascot database searches of the MS and MS/MS spectra, a TonB-dependent receptor, porin and peroxidase were found to be significant and major components of the most abundant excised bands (bands no. 1, 2 and 10, respectively) in the protein profile of TBT-grown cells. These proteins might be involved in organotin resistance.

**Table 2:** Effect of the presence of TBT on the selected proteins of *Pseudomonas* sp. B-219

Band nb^a^	Protein name	Accession no.	Score	Coverage (%)	MW(Da)	TBT effect on protein^b^
1T	TonB-dependent receptor	gi|566065532	330	15	93,368	+
2C	TonB-dependent receptor	gi|589145305	441	15	77,019	
2T	TonB-dependent receptor	gi|589145305	1325	32	77,019	+
3T	60 kDa chaperonin	gi|259585912	1435	38	56,931	+
4C	Aldehyde dehydrogenase	gi|566065968	520	33	54,954	
4T	Aldehyde dehydrogenase	gi|566065968	767	40	54,954	
5T	Succinate–semialdehyde dehydrogenase	gi|589144980	440	37	51,467	+
6T	Porin	gi|566065415	292	24	49,574	+
7T	Membrane protein	gi|566060805	331	18	46,422	+
8C	Succinyl-CoA ligase [ADP-forming] subunit beta	gi|259511788	372	31	41,262	−
9C	Putrescine/spermidine ABC transporter substrate-binding protein	gi|499376521	453	14	40,129	−
10C	glyceraldehyde-3-phosphate dehydrogenase	gi|566065529	822	39	36,125	−
10T	Outer membrane porin F	gi|585710	303	27	34,461	+
11C	ABC transporter	gi|499375547	599	26	32,993	−
11T	Porin	gi|566065186	136	13	34,368	+
12C	LacI family transcriptional regulator	gi|566064643	377	23	32,302	−
13C	Amino acid ABC transporter	gi|566060821	550	31	27,692	−
14C	Peroxidase	gi|566061831	550	57	21,875	
14T	Peroxidase	gi|566061831	569	69	21,875	+
15C	Alkylhydroperoxidereductase subunit C	gi|589147483	147	27	20,496	
15T	Alkylhydroperoxidereductase subunit C	gi|566063546	455	41	20,452	+

^a^C-control sample, T-TBT sample

^b^+ increased abundance, − decreased abundance

On the other hand, the control samples contained proteins involved in amino acid transport (Fig. 5), and the intensity decreases in TBT cultures.

## Discussion

TBT is one of the most toxic xenoestrogens introduced to the environment by humans. It is said that 2 ng l^−1^ disturbs the endocrine system in molluscs (Ruiz et al. 1998). Therefore, we decided to use the microorganism which was able to survive in a culture with a high content of TBT, as a suitable model for the study of organotin-resistance mechanism(s). Similarly to other TBT-degrading microorganisms (*P. diminuta*), the biocide transformation was observed during microbial growth (Kawai et al. 1998). Unfortunately, the only detected TBT metabolite was dibutyltin (DBT). Moreover, significant inhibitory effect of TBT on the growth rate of *Pseudomonas* sp. B-219 was correlated with an increase in the xenoestrogen concentration used in this study. Also, *Pseudomonas aeruginosa* strain 25 W, isolated from surface waters of the Arabian Sea, off the shores of Goa, India grew at the TBT concentration of 50 µM similar to that in the control, but an exposure to 500 μM resulted in a visible decrease in bacterial growth (Dubey et al. 2006).

Changes in the lipid composition of bacteria in response to membrane-active compounds (e.g. phenol, toluene, chlorophenols) are a well known phenomenon (Weber and de Bont 1996; Dercová et al. 2004). TBT, which is a xenoestrogen of high lipophilicity (Martins et al. 2005), is a compound which disturbs lipid homeostasis in Eukaryotic cells as well (Bernat et al. 2013). On the other hand, information about interactions between the organotin and lipids of TBT-resistant bacteria are scarce. Therefore, according to our knowledge, for the first time, changes were observed in the phospholipid profile of bacteria in the presence of TBT. In contrast to eukaryotic cells, the examined cells did not contain phosphatidylcholine (PC) and phosphatidylinositol (PI). This has also been observed in other *Pseudomonas* species, so the absence of PC and PI is not rare (Rühl et al. 2012). Examination of the phospholipid composition of *Pseudomonas* B-219 strain indicates that bacteria can exhibit complex response mechanisms in membrane lipids to combat the effect of TBT. PE and PG are major lipid components of bacterial membranes (Murzyn et al. 2005). In solvent-tolerant bacteria, the amount of PG in relation to PE increases or decreases, depending on the chemical character of the solvent molecules (Weber and de Bont 1996). This alteration in the headgroup composition allows preservation of the stability and low permeability of the membrane by increasing the average phospholipid headgroup area and presumably the chain order (Murzyn et al. 2005). Comparing the results obtained, it can be seen that the most significant changes in *Pseudomonas* sp. B-219 were connected with PG decrease (twofold). According to Murzyn et al. (2005), along with the increasing PG/PE ratio the membrane becomes less permeable for lipophilic and polar molecules. However, in the strain examined, we noted a decreased PG/PE ratio.

The changes in DBI observed within bacterial cultures were mainly caused by an increased content of the less saturated species in TBT-treated samples (Table 1). Higher DBI indicates lower saturation of fatty acids. It seems, that decreased PL unsaturation in TBT exposed *Pseudomonas* sp. leads to a decrease in membrane fluidity of the examined strain (Turk et al. 2004).

Several studies have shown that the generation of reactive oxygen species (ROS) is a major mechanism of TBT toxicity (Gupta et al. 2011; Zhang et al. 2012). On the other hand, it is thought that the catalase/peroxidase subfamily provides protection from oxidative stress in bacteria (Nikodinovic-Runic et al. 2009). Therefore, the up-regulation of peroxidases in *Pseudomonas* sp. B-219 cells could be involved in bacterial resistance towards TBT.

In the organotin-treated sample an increase in the relative abundance of porins was observed. It seems that TBT influenced the selective permeability properties of the bacterial outer membranes which are largely controlled by the porin pool.

Other proteins, whose amounts increased in the presence of TBT were TonB-dependent receptors. TonB-dependent outer-membrane receptor proteins, are responsible for the specific uptake of each ferric-siderophore complex in the bacterial cell (Mirus et al. 2009). However, their function in the cells exposed to TBT is not clear. On the other hand, it is worth pointing out, that pyoverdin (PVD), which was produced by *Pseudomonas chlororaphis* and pyochelin (PCH), a siderophore secreted by *Pseudomonas aeruginosa* were capable of decomposing organotin compounds (Sun and Zhong 2006).

## Conclusion

The examined strain, when exposed to TBT, showed significant changes in the phospholipid concentration and composition, which may reflect the degree of *Pseudomonas* sp. B-219 tolerance to TBT and suggest that this mechanism is utilized to maintain optimal lipid ordering in the presence of the organotin. Moreover, induction of some proteins in the presence of TBT could also play a role in the mechanisms of resistance to oxidative stress-inducing agents. However, it is not possible to provide a full explanation of bacterial membrane response towards TBT. Probably, the *Pseudomonas* B-219 resistance to TBT depends on phospholipid modifications and simultaneous membrane proteins activity. However, the use of 2D-gel electrophoresis in further studies and determination of membrane physical properties, could increase our knowledge concerning microbial resistance to TBT.
